# Microfluidic Raman Sensing Using a Single Ring Negative Curvature Hollow Core Fiber

**DOI:** 10.3390/bios11110430

**Published:** 2021-10-30

**Authors:** Xinyu Wang, Shuguang Li, Shoufei Gao, Yingying Wang, Pu Wang, Heike Ebendorff-Heidepriem, Yinlan Ruan

**Affiliations:** 1 State Key Laboratory of Metastable Materials Science & Technology, Key Laboratory for Microstructural Material Physics of Hebei Province, School of Science, Yanshan University, Qinhuangdao 066004, China; wangxinyu@neuq.edu.cn; 2 School of Computer and Communication Engineering, Northeastern University at Qinhuangdao, Qinhuangdao 066004, China; 3 Institute for Photonics and Advanced Sensing (IPAS), School of Physical Sciences, The University of Adelaide, Adelaide, SA 5005, Australia; heike.ebendorff@adelaide.edu.au; 4 Institute of Photonics Technology, Jinan University, Guangzhou 510632, China; jngaofei@163.com (S.G.); dearyingyingwang@hotmail.com (Y.W.); 5 Institute of Laser Engineering, Beijing University of Technology, Beijing 100124, China; wangpuemail@bjut.edu.cn; 6 School of Electronic Engineering and Automation, Guilin University of Electronic Technology, Guilin 541004, China

**Keywords:** negative-curvature fiber, Raman scattering, fluidic sensing, microfluidic cell

## Abstract

A compact microfluidic Raman detection system based on a single-ring negative-curvature hollow-core fiber is presented. The system can be used for in-line qualitative and quantitative analysis of biochemicals. Both efficient light coupling and continuous liquid injection into the hollow-core fiber were achieved by creating a small gap between a solid-core fiber and the hollow-core fiber, which were fixed within a low-cost ceramic ferrule. A coupling efficiency of over 50% from free-space excitation laser to the hollow core fiber was obtained through a 350 μm-long solid-core fiber. For proof-of-concept demonstration of bioprocessing monitoring, a series of ethanol and glucose aqueous solutions at different concentrations were used. The limit of detection achieved for the ethanol solutions with our system was ~0.04 vol.% (0.32 g/L). Such an all-fiber microfluidic device is robust, provides Raman measurements with high repeatability and reusability, and is particularly suitable for the in-line monitoring of bioprocesses.

## 1. Introduction

Raman spectroscopy is commonly utilized to provide molecular fingerprint information that enables analytes of interest to be identified with the advantages of superior spatial resolution, specificity, and label-free analysis [1]. It has shown potential value for both qualitative and quantitative identification and analysis of a wide range of analytes and is especially suitable for the on-line detection of bioprocesses such as cell culture and microbial fermentation, which benefit from the weak Raman signals of water [2]. By monitoring the concentration change of the nutrients (carbohydrates such as glucose, vitamins, amino acids, minerals, etc.) and side products (such as ethanol and lactate) in the cell culture medium, the culture environment can be improved by feeding suitable chemicals, and thus the quality and amount of end products can be optimized. Conventional Raman probes based on solid-core fibers and bulky optics are widely used for this purpose; however, their limit of detection and high volume demand have restricted their applications in some bioprocesses [3]. In the production process of *Saccharomyces boulardii*, which is widely applied in the fields of animal husbandry and human healthcare products, the concentration level of ethanol needs to be always maintained at a low value of 0.35 vol.% in order to avoid the unnecessary consumption of nutrients including sugars and glucose and the inhibition of yeast growth [4]. It is challenging to achieve such a limit of detection using conventional probes. Therefore, Raman enhancement techniques such as surface-enhanced Raman spectroscopy (SERS) and hollow-core-fiber-enhanced Raman spectroscopy (FERS) have emerged as powerful tools for such applications [5].

In SERS, Raman enhancement is achieved by adsorbing molecules on metal surfaces in nano-structured films or nano-particle suspensions [6,7], which is not suitable for in-line monitoring of variable concentrations of chemicals in bioprocesses. In addition, although SERS has been intensely investigated for 30 years, it is not commercially available yet due to its limited quantitative measurements capability [8].

FERS uses a hollow-core fiber (HCF) as an enhancement platform to provide both a fluid chamber and a high light-analyte interaction, which are inaccessible by conventional fiber-based Raman probes [9,10]. Since light is confined within the central hollow core of the HCF, only a small percentage of light overlaps with the fiber material. Raman interference from silica is thus significantly reduced compared to solid-core fibers. This makes HCFs superior for spectroscopic detection [11], particularly for Raman detection. The first generation of HCFs consists of metal-lined hollow waveguides, which allow enhancing the collection efficiency of Raman signals for liquids/gases filled into the hollow core with the aid of a metal reflector coated inside the fibers’ inner wall [12]. An enhancement by a factor of two was achieved by introducing such a metal-lined hollow waveguide, with a limit of detection (LOD) of 5 vol.% reached for ethanol solutions [13]. However, the metal-lined hollow waveguide presents a high bend loss with limited available length due to its cumbersome metallic coating, which restricts its applicability. An alternative are hollow-core photonic bandgap fibers (PBGFs) [14], which provide a microfluidic channel, a lower loss, and a longer fiber length compared to metal-lined hollow waveguides. PBGFs are widely used to develop temperature sensors, humidity sensors, pressure sensors, gas sensors, as well as fluidic sensors [15,16]. Han et al. fabricated a liquid-core PBGF for solution measurement by selectively collapsing its cladding with the aid of a fusion splicer, where only the central core was utilized as a Raman sensing platform and an LOD of 1 vol.% for ethanol in water was obtained by an index guiding mechanism [17]. The first quantitative Raman detection of glucose at a concentration in the range from 1 mM/L to 25 mM/L was performed by using a liquid-filled PBGF with a coupled laser power of 2 mW and a free space objective [18]. Both selectively and fully filling PBGFs have attracted interest and have rapidly developed in the fiber optic sensing field, but several technical challenges have risen since they were proposed more than 20 years ago. Some features seem to restrict further PBGF application, e.g., (i) their relative complicated cross section structure, (ii) their narrow transmission window (usually around 200 nm of spectral width), (iii) their requirement for fusion or blocking techniques in order to seal the cladding and leave the central core open (for selectively filling cases), and (iv) the limited specific range of the refractive index of samples to be tested (has always to be higher than the index of the fundamental space-filling mode of the cladding when fully or selectively filling occurs so as to guide the total internal reflection mechanism and obtain a broad transmission band) [19].

The recently developed negative-curvature hollow-core fibers (NCFs) have attracted growing attention due to their broader transmission bandwidth and lower transmission loss compared to traditional PBGFs [20,21]. Among these, the single-ring NCF is more convenient for practical applications than the Kagome-lattice fiber, as it takes advantage of the negative curvature of the core boundary, a low transmission loss, as well as a simple cladding structure [22,23,24,25]. It has been recognized that a negative-curvature core shape contributes to reducing the confinement loss by one or two orders of magnitude [26], and efforts in designing and fabricating such NCF have been made by some research groups over the past few years. An efficient delivery of high-energy nanosecond and picosecond pulsed laser light in a single ring NCF was achieved with attenuation of 0.15 dB/m and 0.18 dB/m at 532 nm and 515 nm, respectively, providing a single mode and stable transmission of the source laser beam [27]. Several ultralow-loss single-ring NCFs were designed and fabricated, including one with a transmission loss of 7.7 dB/km at 750 nm and another guiding an ultrabroad band in the ultraviolet, visible, and near-infrared ranges with a loss around 10–20 dB/km, covering from 600 to 1200 nm [28]. The effect of the design flexibility of nested NCFs on the optical characteristics in the visible and near-infrared spectral regime was reported to extend the bandwidth and lower the transmission loss. A wide bandwidth covering from 0.65 μm to 2.5 μm was proposed, and a novel design consisting in applying a lateral cut on the side of the fiber allowed analytes being easily filled, increasing the device’s potential in sensing application [29]. A new kind of conjoined-tube NCF structure, that was designed to conjoin twin anti-resonant tubes in the cladding region to efficiently confine light, achieved a milestone in transmission loss reduction, reaching a minimum of 2 dB/km at 1512 nm. Its bandwidth, covering from 1302 nm to 1637 nm, single modedness, and simple geometry make this NCF a candidate for future optical high-capacity communication systems [30]. In fact, it can be a low-loss waveguide capable of supporting transmission in a large wavelength range, which can promote its application in the sensing field. Research on strain and temperature sensing used an NCF as a microcavity, with the sensitivity of 2.3 pm/με and 1.3 pm/°C, respectively. The use of splicing has featured different interferometric paths and has simplified the manufacture of sensing devices with only one NCF and a fusion splicer, offering an alternative for the development of compact optical sensors [31]. An overall review regarding the recent progress of anti-resonant hollow-core fibers (NCF) for sensing applications in biomedical, biochemical, and environment detection was carried out, illustrating the advantages of such fibers on optofluidic sensing, attributed to their low transmission loss, broad bandwidth, as well as high light–analytes interaction [32]. A proof-of-concept study on Raman detection of pure ethanol under different input powers from 8 mW to 60 mW was presented, paving the way to the application of such NCFs for biochemical sensing analysis [33]. Although a Raman probe with ultra-low background interference was proposed for endoscopic sensing by delivering the pump light through an NCF [34], the prominent advantage of the fiber as a signal enhancement chamber has not been reported yet.

This paper presents a compact and robust microfluidic silica NCF system for the sensitive Raman detection of ethanol and glucose as model analytes for proof-of-concept demonstration. To allow both the excitation laser and the liquid to reach the hollow core of the NCF, at the input port of the NCF, a short piece of solid-core multimode fiber (SCF) (350 μm of length) was used to couple light into the NCF by assembling the two ends of the two fibers into a ferrule. The ends of the SCF and NCF were assembled maintaining a small gap between them to allow pumping of the liquid into the NCF through a side hole of the ferrule in the middle of the gap. The thus created microfluidic cell led to a coupling efficiency higher than 50% with low background Raman interference from the fiber material of the SCF and fast flowing of the liquid into the holes of the NCF. At the output port of the NCF, another ferrule was used to allow the liquid inside the NCF to flow out. Using a backward detection configuration, the LOD of an ethanol solution as low as 0.04 vol.% (0.32 g/L) was achieved, which demonstrates that this system has potential application for the on-line determination and remote monitoring of analytes in bioprocesses.

## 2. Numerical Analysis

**Figure 1:**
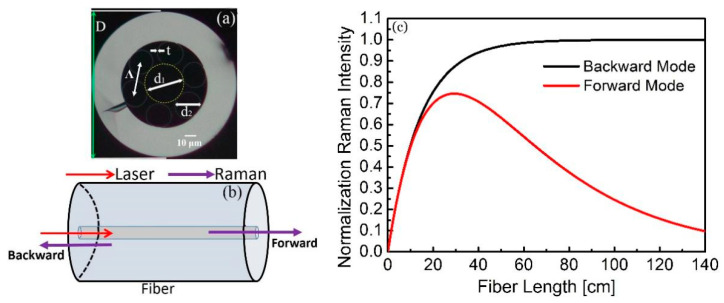
(**a**) Cross-sectional image of the NCF and configurations of (**b**) two collection geometries and (**c**) their relative Raman intensity dependence on NCF length.

An optical image of the cross section of the silica-based NCF is presented in Figure 1a. The NCF had an outer diameter of 125 μm. The hollow core diameter of ~32 μm is defined as the yellow circle inscribed by the single-layer cladding. The cladding was composed of six separate thin tubes with air hole diameter of 20 μm and wall thickness of 250 nm; its lattice constant *Ʌ* = *d*_1_/2 + *t* + *d*_2_/2 was 26.25 μm. The inhibited light coupling between the core and the cladding regions confined the light in the low-refractive-index (RI) hollow-core region at the wavelengths that satisfied the anti-resonant condition [35]. By analogy to one-dimensional slab waveguides, the anti-resonant condition in NCFs occurs when [36]:

(1) $$\frac{2\pi t}{\lambda }\sqrt{n_{glass}^{2}-n_{air}^{2}}=k_{0}t\sqrt{n_{glass}^{2}-n_{air}^{2}}=\left(m-0.5\right)\pi ,\;m=1,\;2,\;3\ldots$$

where *t* represents the wall thickness of the cladding tubes, *n_glass_* and *n_air_* are the RI of the tube material and air, respectively, and *λ* and *k*_0_ = 2π⁄*λ* indicate the operating wavelength and the corresponding wavenumber, respectively.

In this section, the collection efficiency of the Raman signals by the NCF is calculated, and its dependence on the length of the fiber is explored. As shown in Figure 1b, Raman signals can be usually collected either from forward direction or backward direction. A numerical model was developed to investigate the dependence of the Raman signal power on these two collection schemes, as well as the length of the fiber, simplifying the generated Raman power, *P*_R_, using the following expression [37]:

(2) $$P_{R}=\frac{P_{L}s\pi NA}{n_{\mathrm{co}}^{2}}z_{e}$$

where *P*_L_ is the laser power launched into the fiber, *s* is the Raman scattering coefficient that is determined by the Raman cross section of the analyte, *NA* is the numerical aperture of the fiber, which determines the coupling efficiency from the laser into the fiber, *n*_co_ indicates the RI of the fiber core, and *z*_e_ denotes the effective fiber length. This equation shows that the Raman power is proportional to the effective fiber length *z*_e_ for both the forward and the backward collection schemes; thus, the relative magnitude of the Raman power can be predicted by considering:

(3) $$z_{e}=\int_{z=0}^{z=z_{p}}T_{L}\left(z\right)\cdot T_{R}\left(z\right)dz$$

where *T*_L_(*z*) and *T*_R_(*z*) describe the propagation characteristics of the laser and the Raman signals, which are related to the corresponding attenuation coefficients *α* of light, and *z*_p_ indicates the real length of the fiber. Using the loss coefficients of the fiber itself, *α*_L,1_ and *α*_L,2_, and the absorption coefficients *α*_R,1_ and *α*_R,2_ of the analyte at the excitation laser and Raman wavelengths, respectively, the evolution of *z*_e_ can be considered as follows for the liquid analyte-filled NCF [37]:

(4) $$z_{e}=\frac{e^{-\alpha_{L}z_{p}}-e^{-\alpha_{R}z_{p}}}{\alpha_{R}-\alpha_{L}}\;\;\;\mathrm{Forward}\;\mathrm{scattering}$$

(5) $$z_{e}=\frac{1-e^{-\left(\alpha_{L}+\alpha_{R}\right)z_{p}}}{\alpha_{L}+\alpha_{R}}\;\;\;\;\;\;\mathrm{Backward}\;\mathrm{scattering}\;$$

where *α*_L_ = *α*_L,1_ + *α*_L,2,_ and *α*_R_ = *α*_R,1_ + *α*_R,2_. The value of *z*_e_ for our NCF was calculated using the following parameters: excitation wavelength of 785 nm, Raman frequency ranging from 0 cm^−1^ to 2020 cm^−1^ (corresponding to 785 nm–933 nm for the 785 nm wavelength excitation). The absorption coefficients for the low-concentration mixed solutions of ethanol and water were approximately determined on the basis of that of pure water, which are 2.5 dB/m at the excitation wavelength (785 nm) and 4.5 dB/m at the Raman frequency (933 nm) [38,39]. The confinement loss of the NCF was analyzed utilizing the COMSOL Multiphysics software, and the 0.0077 dB/m and 0.027 dB/m at 785 nm and 933 nm were numerically calculated for the NCF fully filled with the mixed solutions. It is clear that the confinement loss values were about three orders of magnitude smaller than the absorption of the mixed solutions, illustrating that the infiltration of the analytes had a negligible influence on the total attenuation of the NCF, but the absorption effects of the analytes significantly affected the collection efficiency of the Raman signal.

Figure 1c shows the normalized results for the forward and backward collection efficiency as a function of the fiber length *z*_p_. It can be seen that the forward collected Raman intensity reached the maximum at the fiber length of ~30 cm and then gradually decreased to zero due to fiber loss and water absorption. The backward collected Raman intensity increased steeply with the increase of the fiber length and then saturated when the fiber length was ~50 cm. The maximum Raman intensity for the backward collection was ~30% higher than that for the forward collection. Thus, the backward collection scheme was adopted in our experimental setup.

Equation (2) also illustrates the effect of the laser power *P*_L_ and the *NA* of the NCF on the Raman power *P*_R_. Increasing *P*_L_ is a straightforward way to enhance the Raman signal of analytes. A high-index glass such as germanate for the fiber material is also helpful to increase the *NA*, thus enhancing the Raman power.

## 3. Experiment and Results

### 3.1. Microfluidic Cell Design and Optimization

**Figure 2:**
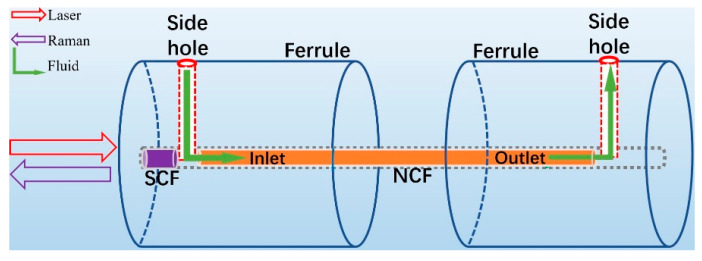
Schematic chart of the proposed microfluidic cell.

To realize both high-efficiency optical coupling and continuous filling of the liquid into the holes of the NCF in real-time Raman measurements, a specialized microfluidic cell was designed (Figure 2). Parry et al. proposed a PBGF gas sensor with a similar fiber assembly design, which was glued to the surface of a hollow aluminum cylinder with a height of 160 mm [40]. In Figure 2 shows our 1 cm-long microfluidic cell obtained using a very simple and compact assembly process and consisting of a ceramic ferrule with a mechanically drilled side hole for pumping the liquid into the central channel of the ferrule. Within the ferrule, an SCF (core diameter of 25 μm, numerical aperture of 0.1) and the NCF were assembled maintaining a gap between them to allow for the liquid to be pumped through the side hole of the ferrule into the holes of the NCF. The output end of the NCF was placed into another ferrule with a side hole enabling the analyte to flow out. In this assembly, the SCF acts not only as a medium to couple the laser light into the NCF, but also as an indispensable plug to contain the liquid within the cell. Our microfluidic cell design allows any analyte of interest to be easily pumped into the NCF by means of a syringe and a pump, providing a constant injection speed and avoiding the formation of air bubbles.

**Figure 3:**
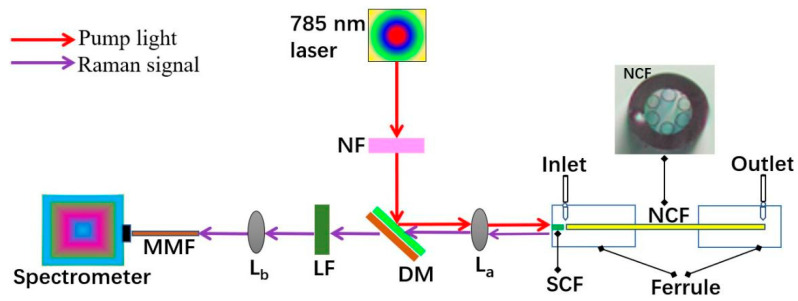
Schematic of the experimental setup of our Raman detection system. (L_a_ and L_b_ are molded lenses. NF, DM, and LF represent notch filter, dichroic mirror, and long pass filter, respectively).

The schematic of the experimental setup for our Raman detection system is presented in Figure 3. A 785 nm CW single-mode diode laser with 125 mW power passed through a notch filter (NF) and was reflected by a dichroic mirror (DM) to a molded borosilicate glass lens L_a_. The laser was first coupled into the SCF by the lens and then into the NCF within the ferrule. The backward scattered light was collimated by the lens and then passed through the DM and a 785 nm-long pass filter (LF) to remove the Rayleigh scattering of the 785 nm source. Finally, the backward scattered Raman signal was coupled into a multimode fiber (MMF) via another molded lens, L_b_, and recorded with a spectrometer.

**Figure 4:**
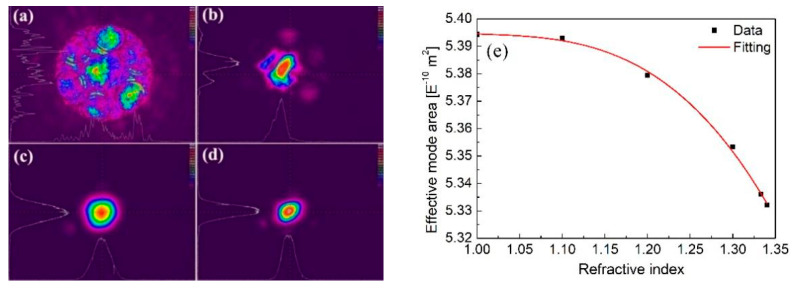
Mode field distributions for the (**a**) hollow NCF and (**b**) the water-filled NCF. The pump wavelength was 532 nm. Mode field distributions for the (**c**) hollow NCF and (**d**) the water-filled NCF. The pump wavelength was 785 nm, (**e**) mode area as a function of the RI of the analytes non-selectively introduced into the NCF.

The mode images of the NCFs with and without water filling at 532 nm and 785 nm are shown in Figure 4. It can be seen that for the wavelength of 532 nm, when the fiber was not filled with water (Figure 4a), the light strongly leaked into the cladding rings, and only a small percentage of the light was confined in the hollow core (RI = 1.0); in contrast the light was mostly confined in the core region when the fiber was fully filled with water (RI = 1.333) (Figure 4b). The unfilled fiber was not expected to guide light through its core at a wavelength of ~530 nm with cladding tubes thickness of 250 nm. For the wavelength of 785 nm, the NCF showed single-mode characteristics both with and without water filling (Figure 4c,d). The mode area even became slightly smaller and more confined when the NCF was fully filled with water. Figure 4e illustrates the numerical simulation results of the effective mode area as a function of the RI of the analytes introduced into the NCF, and shows that the mode area slightly decreased as RI increased. This phenomenon could be investigated experimentally (Figure 4c,d). Although the molecules to be measured usually show stronger Raman signals at short wavelength [41], stronger background fluorescence interference commonly exists at short wavelength during on-line monitoring of biomaterials. Thus, a 785 nm laser was selected as the excitation source to explore the Raman detection capability of the NCF in our experiments.

In the design of the microfluidic cell described above, a piece of step-index silica SCF was used to couple the laser into the NCF. However, such an SCF may produce strong background Raman signals dependently on the fiber length. Therefore, the effect of the length of the SCF on the Raman signals collected by the NCF through the backward scattering scheme was examined. An SCF with a core diameter of 25 μm and an NA of 0.1 was chosen and matched to the NCF to ensure low coupling loss between them. SCFs with different lengths of 95 mm, 50 mm, 5.5 mm, 0.5 mm, and 0.35 mm were separately butt-coupled to an NCF of 20 cm length within a ferrule. For SCF pieces with lengths longer than 5.5 mm, the gap between the SCF and the NCF was located in the middle position along the radial direction of the ferrule. For other SCFs with shorter lengths, the position of the gap was adjusted to allow the whole length of the SCF to be located inside the hole of the ferrule. They were assembled with a gap of about 50 μm, and the position of the side hole of the ceramic ferrule was also adjusted to match the position of the gap between the SCF and the NCF to enable pumping the liquid into the gap and then into the NCF. This was controlled by drilling the side hole of the ceramic ferrule in a position close to the end surface of the ferrule. Both the SCF and the NCF were glued first, and their accurate positions were aligned under an optical microscope. After the glue was dry, the SCF was cut at the end of the ceramic ferrule and then polished for laser coupling from the SCF to the NCF. The assembled structure provided a smooth end face of the SCF, allowing a high laser coupling efficiency of nearly 90% to the SCF. The coupling efficiency from the SCF to the unfilled NCF with a gap ~50 μm was over 50% and decreased to ~25% when the NCF was filled with liquid such as water, due to the changed mode field distribution of the NCF and the absorption of the liquid. However, this coupling efficiency was still comparable with that of the conventional laser free-space coupling scheme based on a lens coupling to the HCF enclosed within a glass chamber allowing the liquid to flow in and out [42]. The conventional free-space coupling structure is difficult to assemble and has a high cost. Our simple design and assembly to integrate light coupling and microfluidic cell through a cheap ferrule is easy to build, achieves repeatable coupling efficiency, has a largely reduce cost, and is robust and able to sustain any mechanical impact since it does not include any bulk glass structure.

**Figure 5:**
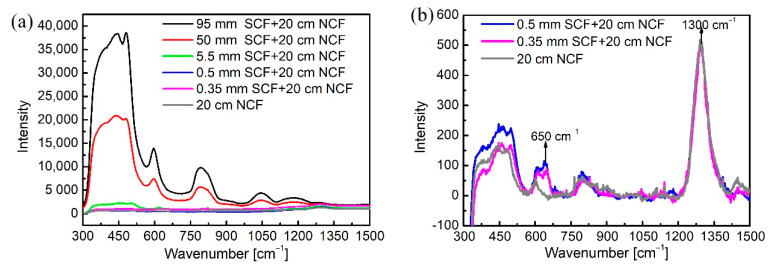
Background Raman signal for different lengths of SCF butt-coupled to a 20 cm-long unfilled NCF. (**a**) Spectra for SCF with lengths from 95 mm to 0.35 mm and the 20 cm-long NCF. (**b**) Specific spectra for SCF with lengths from 0.5 mm to 0.35 mm and the 20 cm-long NCF corresponding to (**a**).

The Raman spectra corresponding to different lengths of the SCF butt-coupled to the 20 cm-long unfilled NCF are shown in Figure 5, with the integration time and the input power being kept constant to 10 s and 125 mW, respectively. The Raman spectra of the 20 cm-long unfilled NCF without the SCF is also shown for clear comparison. Figure 5a presents the typical Raman profile of silica (e.g., the black and red lines), illustrating that any signals from the analytes would be overwhelmed under this strong background interference, as the SCF is tens of millimeters long. Figure 5b resolves Figure 5a by showing SCFS with shorter lengths of 0.5 mm and 0.35 mm as well as a single unfilled NCF without coupling with the SCF; the peaks with constant intensity at 650 cm^−1^ and 1300 cm^−1^ from the ferrule and molded lens, respectively, are visible. All the other Raman peaks correspond to the silica material of both the SCF and the NCF. It was found that, when the length of the SCF decreased from 95 mm to 0.35 mm, the silica Raman intensity was reduced by about 200 times, comparable with that of the single NCF itself examined in the setup. This result demonstrates that the signal from the SCF of 0.35 mm length was negligible. Considering the feasibility of the lateral drilling technique for the ferrule and the remarkable reduction of background interference, a 0.35 mm-long SCF was used for the experiments with ethanol solutions.

### 3.2. Quantitative Detection of Ethanol Solutions

**Figure 6:**
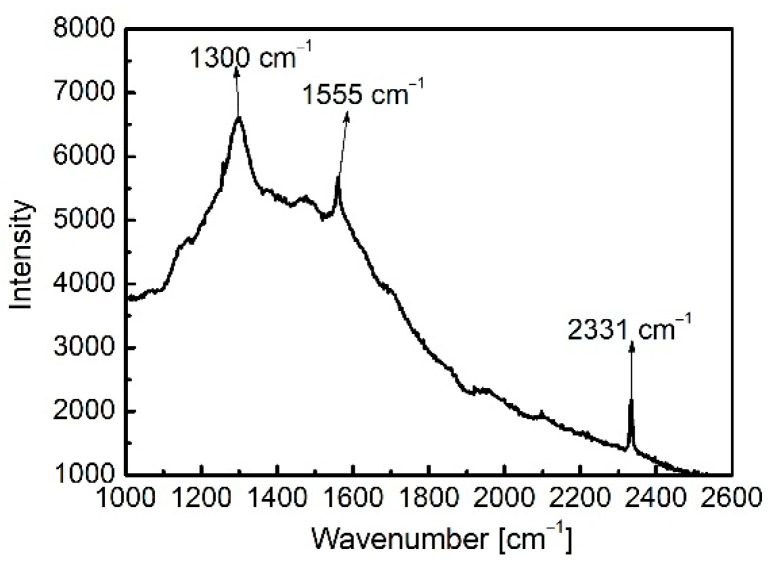
Raman response of oxygen (1555 cm^−1^) and nitrogen (2331 cm^−1^) molecules in the air present in the hollow NCF. (The peak at 1300 cm^−1^ originates from the molded lens L_a_ in the configuration).

Prior to the quantitative detection of ethanol solutions, the Raman response of the unfilled NCF with a length of 80 cm was firstly measured, as shown in Figure 6, where the integration time was 10 s, and the output power was about 64 mW. Raman peaks at 1300 cm^−1^, 1555 cm^−1^, and 2331 cm^−1^ are observed, and it can be concluded that the intrinsic peak at 1300 cm^−^^1^ originated from the borosilicate glass molded lens L_a_ in the configuration [43]. The other two peaks of 1555 cm^−1^ and 2331 cm^−1^ correspond exactly to the characteristic Raman lines of oxygen (O_2_) and nitrogen (N_2_), respectively [44], illustrating that O_2_ and N_2_ molecules in the low-volume ambient air existing in the micro-hole of the unfilled NCF could be detected, which shows the sensing potential of our design.

**Figure 7:**
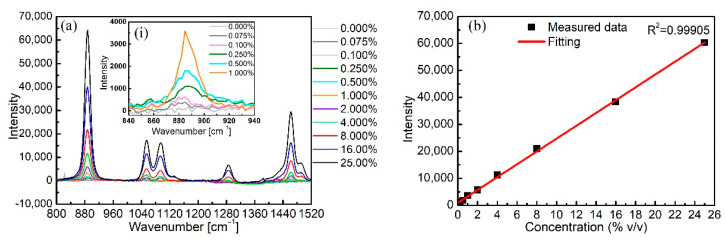
(**a**) Raman spectra of ethanol solutions at different concentrations (from 0 vol.% to 25 vol.%) and (**b**) the corresponding linear fitting curve.

Then, the NCF was cut into a 30 cm-long piece for Raman signal measurements of a series of aqueous ethanol solutions, using the Raman signal of the pure water-filled NCF as the background reference. Solutions with increased ethanol concentrations were sequentially introduced into the same NCF, and the corresponding Raman spectra were recorded using the same measurement conditions of 125 mW input power, 0.001 mL/min pump rate, and 10 s integration time. Prior to the injection of a new ethanol solution with different concentration, Milli-Q water was injected to completely remove the previous solution. The injection of the solution was paused once the NCF was fully filled. Note that only 3.7 μL of solution was needed to completely fill the fiber, and all measurements were repeated three times. Figure 7a presents the evolution of the Raman signals of the ethanol solutions with concentrations increasing from 0 vol.% to 25 vol.%. For all measured Raman spectra data, the background spectrum of pure water was subtracted. Characteristic Raman peaks of 884 cm^−1^, 1055 cm^−1^, 1090 cm^−1^, 1280 cm^−1^, and 1460 cm^−1^ for pure ethanol can be seen in the Figure. Each peak intensity increased with the increasing ethanol concentration as expected, and the peak at 884 cm^−1^ with the highest intensity is regarded as a fingerprint Raman signal of ethanol, normally used to distinguish it from other analytes as well as to obtain an LOD. The inset in Figure 7a is an enlarged view of the peak at 884 cm^−1^ to better observe the variation of peak intensity at lower concentrations of ethanol from 0 vol.% to 1 vol.%; the dependence of its intensity on ethanol concentration is illustrated in Figure 7b, where an excellent linearity between the intensity and the concentration is demonstrated. The LOD of ethanol was calculated as the concentration corresponding to the sum of the mean and three times the standard deviation of the background Raman signal of pure water at 884 cm^−1^ [45]; its value was determined to be 0.04 vol.% (0.32 g/L).

### 3.3. Quantitative Detection of Glucose Solutions

**Figure 8:**
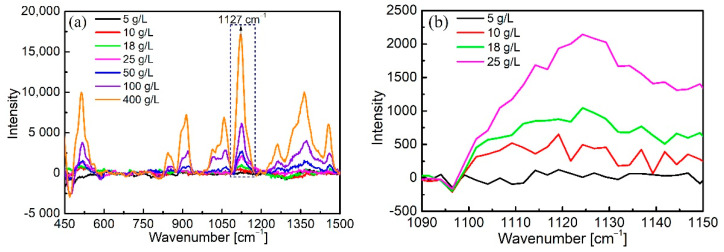
(**a**) Raman spectra of glucose solutions at different concentrations and (**b**) spectra of glucose solutions at lower concentration.

The measurement of glucose solutions was then carried out following the same procedure described above. A series of glucose solutions at concentrations from 5 g/L to 400 g/L were prepared and pumped into the assembly, separately. The characteristic Raman peaks of glucose including 511 cm^−1^, 1065 cm^−1^, and 1127 cm^−1^ peaks can be observed in Figure 8a. The intensity of the strongest peak at 1127 cm^−1^ was selected as the specific peak for quantitative monitoring, and its intensity at lower concentrations is presented in Figure 8b. The Raman peak intensity increased with increasing glucose concentration. For the concentration of 5 g/L, the peak was almost invisible, which suggests that the detection limit of glucose was in the order of 10 g/L. This value is roughly 20 times lower than the LOD of ethanol and is not consistent with the results obtained by conventional Raman spectroscopy [46]. Further investigations will be conducted to find the reasons for this difference and how the detection limit can be enhanced.

## 4. Discussion

A brief comparison of our results with others reported recently is shown in Table 1. Compared to other methods to collect the Raman signals of ethanol solutions (free-space or metal-lined hollow waveguides) [12,47], the LOD we achieved was reduced by ~125 times. Our design also provides a natural microchannel for fast liquid injection and strong light-matter interaction that is more suitable for probe sensing than the free-space device. Such an LOD, together with the linear function between the concentration and the Raman intensity, will enable the quantitative monitoring of fermentation processes to ensure a highly efficient production of biomolecules. Glucose and ethanol are two important chemicals to be measured in the process of microbial fermentation. However, it is necessary to further improve the sensitivity of the NCF system to enable the sensing of glucose at concentrations as low as 3 g/L, since this LOD value is required by the fermentation industry [48]. It means that the currently achieved minimum concentration of 5-10 g/L for glucose needs to be reduced by at least a factor of 2.

Possible solutions to increase the sensitivity of our system include the reduction of background Raman interference from the SCF by modifying the ferrule and the soft glass lens used in our setup. It is possible to further reduce the length of the SCF to 100 μm by lateral drilling the ferrule using a more precise laser machining technique. By further reducing the width of the gap between the SCF and the NCF within the ferrule, the percentage of the light directed to the internal surface of the ferrule can be reduced to weaken the Raman signals from the ferrule. The borosilicate glass lens used in our experiments can be replaced by a silica lens to eliminate its Raman interference peak at 1300 cm^−1^. In addition, our numerical modeling for the collection efficiency of Raman signals indicates that the pump laser power and the numerical aperture of the NCF have positive effects on Raman intensity. Thus, the easiest method to increase the sensitivity is to increase the power of the laser, which involves a much higher cost; a high-index-glass-based NCF also has the potential to increase the Raman intensity. Considering that the excitation wavelength is usually in the visible/NIR range, a germanate glass seems a good choice.

## 5. Conclusions

We demonstrated that a single-ring negative-curvature hollow core-fiber has the capability to detect ethanol and glucose in solution at low concentrations through Raman spectroscopy; these are common chemicals produced in many microbial fermentation processes. By assembling a simple microfluidic cell structure using a ferrule, a series of aqueous solutions with increased concentration were pumped into the NCF for Raman measurements, achieving a limit of detection for ethanol solutions of 0.04 vol.%. A perfect linear calibration function between the concentration and the intensity makes the proposed Raman detection system a candidate for bioprocess monitoring, such as in the production process of *Saccharomyces boulardii*. By pumping the samples out of the bioreactor and into the NCF, the composition of the fermentation sample can be analyzed in-line.

## Figures and Tables

**Table 1:** Brief comparison of Raman detection by different methods.

Configuration Type	LOD of Ethanol	Ref.
Free space	4.76%	[47]
Metal-lined hollow waveguide	5 vol.%	[12]
Negative-curvature hollow-core fiber	0.04 vol.%	[our work]

## References

[1] Zheng W., Zhu Y.M., Li F.D., Huang F. (2018). Raman spectroscopy regulation in van der Waals crystals. Photonics Res..

[2] Carey D.M., Korenowski G.M. (1998). Measurement of the Raman spectrum of liquid water. J. Chem. Phys..

[3] Uysal R.S., Soykut E.A., Boyaci I.H., Topcu A. (2013). Monitoring multiple components in vinegar fermentation using Raman spectroscopy. Food Chem..

[4] Zheng G.B., Yu X.F., Li Z.H., Yu M.H., Yao J., Chi B.G., Wang J. (2015). Boulardii Active Dry Yeasts and Production Method Thereof. Chinese Patent.

[5] Frosch T., Yan D., Popp J. (2013). Ultrasensitive fiber enhanced UV resonance Raman sensing of drugs. Anal. Chem..

[6] Wu Y., Jiang T., Wu Z., Yu R. (2018). Novel ratiometric surface-enhanced Raman spectroscopy aptasensor for sensitive and reproducible sensing of Hg^2+^. Biosens. Bioelectron..

[7] Cheng C., Li J., Lei H.X., Li B.J. (2018). Surface enhanced Raman scattering of gold nanoparticles aggregated by a gold-nanofilm-coated nanofiber. Photonics Res..

[8] Suzuki T., Kitahama Y., Matsuura Y., Ozaki Y., Sato H. (2012). Development of a flexible fiber surface-enhanced Raman scattering (SERS) probe using a hollow optical fiber and gold nanoparticles. Appl. Spectrosc..

[9] Tani F., Kotting F., Novoa D., Keding R., Russell P.S.J. (2018). Effect of anti-crossings with cladding resonances on ultrafast nonlinear dynamics in gas-filled photonic crystal fibers. Photonics Res..

[10] Knebl A., Yan D., Popp J., Frosch T. (2018). Fiber enhanced Raman gas spectroscopy. TrAC Trends Anal. Chem..

[11] Yan D., Popp J., Pletz M.W., Frosch T. (2017). Highly sensitive broadband Raman sensing of antibiotics in step-index hollow-core photonic crystal fibers. ACS Photonics.

[12] Liu Y., Wang J., Li Z., Wang J., Ning Y., Liu T., Grattan K.T. (2018). Enhanced Raman Detection System Based on a Hollow-Core Fiber Probe Design. IEEE Sens. J..

[13] Cai H., Yu X., Chu Q., Jin Z., Lin B., Wang G. (2019). Hollow-core fiber-based Raman probe extension kit for in situ and sensitive ultramicro-analysis. Chin. Opt. Lett..

[14] Cregan R.F., Mangan B.J., Knight J.C., Birks T.A., Russell P.S., Roberts P.J., Allan D.C. (1999). Single-mode photonic band gap guidance of light in air. Science.

[15] Arman H., Olyaee S. (2021). Realization of low confinement loss acetylene gas sensor by using hollow-core photonic bandgap fiber. Opt. Quant. Electron..

[16] Li J., Yan H., Dang H., Meng F. (2021). Structure design and application of hollow core microstructured optical fiber gas sensor: A review. Opt. Laser Technol..

[17] Han Y., Oo M.K.K., Zhu Y., Xiao L., Demohan M.S., Jin W., Du H.H. (2008). Index-guiding liquid-core photonic crystal fiber for solution measurement using normal and surface-enhanced Raman scattering. Opt. Eng..

[18] Yang X., Zhang A.Y., Wheeler D.A., Bond T.C., Gu C., Li Y. (2012). Direct molecule-specific glucose detection by Raman spectroscopy based on photonic crystal fiber. Anal. Bioanal. Chem..

[19] Jin W., Xuan H.F., Ho H.L. (2010). Sensing with hollow-core photonic bandgap fibers. Meas. Sci. Technol..

[20] Frosz M.H., Roth P., Gunendi M.C., Russell P.S.J. (2017). Analytical formulation for the bend loss in single-ring hollow-core photonic crystal fibers. Photonics Res..

[21] Yan D., Frosch T., Kobelke J., Bierlich J., Popp J., Pletz M.W., Frosch T. (2018). Fiber-Enhanced Raman Sensing of Cefuroxime in Human Urine. Anal. Chem..

[22] Russell P.S.J. (2006). Photonic-crystal fibers. J. Lightw. Technol..

[23] Yu F., Knight J.C. (2016). Negative curvature hollow-core optical fiber. IEEE J. Sel. Top. Quantum Electron.

[24] Gérôme F., Jamier R., Auguste J.L., Humbert G., Blondy J.M. (2010). Simplified hollow-core photonic crystal fiber. Opt. Lett..

[25] Wang Y.Y., Couny F., Roberts P.J., Benabid F. Low Loss Broadband Transmission in Optimized Core-Shape Kagome Hollow-core PCF. Proceedings of the Conference on Lasers and Electro-Optics, Optical Society of America.

[26] Yu F., Wadsworth W.J., Knight J.C. (2012). Low loss silica hollow core fibers for 3–4 μm spectral region. Opt. Express.

[27] Jaworski P., Yu F., Carter R.M., Knight J.C., Shephard J.D., Hand D.P. (2015). High energy green nanosecond and picosecond pulse delivery through a negative curvature fiber for precision micro-machining. Opt. Express.

[28] Debord B., Amsanpally A., Chafer M., Baz A., Maurel M., Blondy J.M., Hugonnot E., Scol F., Vincetti L., Gerome F. (2017). Ultralow transmission loss in inhibited-coupling guiding hollow fibers. Optica.

[29] Belardi W. (2015). Design and properties of hollow antiresonant fibers for the visible and near infrared spectral range. J. Lightwave Technol..

[30] Gao S.F., Wang Y.Y., Ding W., Jiang D.L., Gu S., Zhang X., Wang P. (2018). Hollow-core conjoined-tube negative-curvature fibre with ultralow loss. Nat. Commun..

[31] Ferreira M.S., Bierlich J., Kobelke J., Pinto J.L., Wondraczek K. (2021). Negative curvature hollow core fiber sensor for the measurement of strain and temperature. Opt. Express.

[32] Ni W., Yang C., Luo Y., Xia R., Lu P., Hu D.J.J., Danto S., Shum P.P., Wei L. (2021). Recent Advancement of Anti-Resonant Hollow-Core Fibers for Sensing Applications. Photonics.

[33] Liu X.L., Ding W., Wang Y.Y., Gao S.F., Cao L., Feng X., Wang P. (2017). Characterization of a liquid-filled nodeless anti-resonant fiber for bio-chemical sensing. Opt. Lett..

[34] Yerolatsitis S., Yu F., McAughtrie S., Tanner M.G., Fleming H., Stone J.M., Campbell C.J., Birks T.A., Knight J.C. (2019). Ultra-low back-ground Raman sensing using a negative curvature fibre and no distal optics. J. Biophotonics.

[35] Wei C., Weiblen R.J., Menyuk C.R., Hu J. (2017). Negative curvature fibers. Adv. Opt. Photonics.

[36] Litchinitser N.M., Abeeluck A.K., Headley C., Eggleton B.J. (2002). Anti-resonant reflecting photonic crystal optical waveguides. Opt. Lett..

[37] Altkorn R., Malinsky M.D., Van Duyne R.P., Koev I. (2001). Intensity considerations in liquid core optical fiber Raman spectroscopy. Appl. Spectrosc..

[38] Wojtanowski J., Mierczyk Z., Zygmunt M. Laser Remote Sensing of Underwater Objects. Proceedings of the SPIE Remote Sensing of the Ocean, Sea Ice, and Large Water Regions, International Society for Optics and Photonics.

[39] Afshar S., Ruan Y., Warren-Smith S.C., Monro T.M. (2008). Enhanced fluorescence sensing using microstructured optical fibers: A comparison of forward and backward collection modes. Opt. Lett..

[40] Parry J.P., Griffiths B.C., Gayraud N., McNaghten E.D., Parkes A.M., MacPherson W.N., Hand D.P. (2009). Towards practical gas sensing with micro-structured fibres. Meas. Sci. Technol..

[41] Yang R., Liu S.P. (2001). Development of some molecular spectral analytical methods for the determination of proteins. Chin. J. Anal. Chem..

[42] Khetani A., Momenpour A., Alarcon E.I., Anis H. (2015). Hollow core pho-tonic crystal fiber for monitoring leukemia cells using surface enhanced Raman scattering (SERS). Biomed. Opt. Express.

[43] Schrader B., Hoffmann A., Keller S. (1991). Near-infrared Fourier trans-form Raman spectroscopy: Facing absorption and background. Spectrochim. Acta. A.

[44] Sutherland G.B.B.M. (1933). Experiments on the Raman effect at very low temperatures. Proc. R. Soc. Lond. A.

[45] Zuber A., Bachhuka A., Tassios S., Tiddy C., Vasilev K., Ebendorff-Heidepriem H. (2020). Field Deployable Method for Gold Detection Using Gold Pre-Concentration on Functionalized Surfaces. Sensors.

[46] Shao J., Lin M., Li Y., Li X., Liu J., Liang J., Yao H. (2012). In Vivo Blood Glucose Quantification Using Raman Spectroscopy. PLoS ONE.

[47] Ye J.F., Wei H.Y., Qi X.H., Li Y., Wang S., Zhao Y., Zou M.Q. (2021). Dual-wavelength Rapid Excitation Raman Difference Spectroscopy System for Direct Detection of Ethanol in Illegal Beverages. Chin. J. Anal. Chem..

[48] Maiorella B.L., Blanch H.W., Wilke C.R. (1983). By-product inhibition effects on ethanolic fermentation by Saccharomyces cere-visiae. Biotechnol. Bioeng..

